# Collectin-K1 Plays a Role in the Clearance of *Streptococcus agalactiae* in Nile Tilapia (*Oreochromis niloticus*)

**DOI:** 10.3390/ijms25052508

**Published:** 2024-02-21

**Authors:** Jinfeng Mo, Jiadong Li, Li Qiu, Yiqing Wang, Liangliang Mu, Jianmin Ye

**Affiliations:** 1 Guangzhou Key Laboratory of Subtropical Biodiversity and Biomonitoring, Institute of Modern Aquaculture Science and Engineering, School of Life Sciences, South China Normal University, Guangzhou 510631, China; 2018010164@m.scnu.edu.cn (J.M.); 2020022757@m.scnu.edu.cn (J.L.); 2021022976@m.scnu.edu.cn (L.Q.); wangyiqing21@hnsfdx.wecom.work (Y.W.); lianglaingmu@m.scnu.edu.cn (L.M.); 2 Guangdong Provincial Water Environment and Aquatic Products Security Engineering Technology, Research Center, Guangzhou Key Laboratory of Aquatic Animal Diseases and Waterfowl Breeding, College of Animal Science and Technology, Zhongkai University of Agriculture and Engineering, Guangzhou 510225, China; 3 Guangdong Provincial Engineering Technology Research Center for Environmentally-Friendly Aquaculture, School of Life Sciences, South China Normal University, Guangzhou 510631, China

**Keywords:** CL-K1, *Streptococcus agalactiae*, Nile tilapia, antimicrobial activity

## Abstract

Collectin-K1 (CL-K1) is a multifunctional C-type lectin that has been identified as playing a crucial role in innate immunity. It can bind to carbohydrates on pathogens, leading to direct neutralization, agglutination, and/or opsonization, thereby inhibiting pathogenic infection. In this study, we investigated a homolog of CL-K1 (*On*CL-K1) in Nile tilapia (*Oreochromis niloticus*) and its role in promoting the clearance of the pathogen *Streptococcus agalactiae* (*S. agalactiae*) and enhancing the antibacterial ability of the fish. Our analysis of bacterial load displayed that *OnCL-K1* substantially reduced the amount of *S. agalactiae* in tissues of the liver, spleen, anterior kidney, and brain in Nile tilapia. Furthermore, examination of tissue sections revealed that *On*CL-K1 effectively alleviated tissue damage and inflammatory response in the liver, anterior kidney, spleen, and brain tissue of tilapia following *S. agalactiae* infection. Additionally, *OnCL-K*1 was found to decrease the expression of the pro-inflammatory factor *IL-6* and migration inhibitor *MIF*, while increasing the expression of anti-inflammatory factor *IL-10* and chemokine *IL-8* in the spleen, anterior kidney, and brain tissues of tilapia. Moreover, statistical analysis of survival rates demonstrated that *On*CL-K1 significantly improved the survival rate of tilapia after infection, with a survival rate of 90%. Collectively, our findings suggest that *On*CL-K1 plays a vital role in the innate immune defense of resisting bacterial infection in Nile tilapia. It promotes the removal of bacterial pathogens from the host, inhibits pathogen proliferation in vivo, reduces damage to host tissues caused by pathogens, and improves the survival rate of the host.

## 1. Introduction

Innate immunity serves as the primary defense mechanism against pathogenic infections [1,2]. During the early stages of infection, innate immune cells and molecules rapidly and specifically initiate innate immune recognition, resulting in the secretion of inflammatory cytokines, enhanced phagocytic cell function, pathogen clearance, and the initiation of adaptive immune responses through the presentation of pathogen antigens [3,4,5,6]. These reactions involve various receptor molecules, such as lectins and toll-like receptors, which are responsible for detecting the presence of causative agents [7,8,9]. Among these receptors, lectins, a group of proteins that bind to carbohydrates, play a vital role in innate immunity by performing multiple functions in host defense [10,11,12,13]. By recognizing and binding to the carbohydrates on pathogenic bacteria, lectins actively become involved in immune responses, including agglutination, complement activation, inflammation regulation, and clearance of causative agents [6,14,15,16,17]. This active involvement helps reduce infection and maintain host homeostasis [6,14,15,16,17].

Collectin Kidney 1 (CL-K1) is a member of the C-type lectin super family. It was discovered in 2006 [18] and has been identified as a pattern-recognition molecule (PRR) of the lectin complement pathway [17]. CL-K1 shares a semblable structure with other C-type lectins, such as mannan-binding lectin (MBL). It consists of a globular head, followed by a neck and a collagenous tail [19]. The head contains a carbohydrate recognition domain (CRD), while the tail contains binding sites for MBL-associated serine proteases (MASPs), which are necessary for complement activation [19,20]. A triplet structure was formed by CL-K1 monomers, which combine to form oligomers with a higher binding affinity for ligands [19]. CL-K1 has been shown to preferentially bind to fucose, mannose, and high-mannose oligosaccharides found on both self- and non–self structures [19,20,21]. The CRD of CL-K1 plays a vital role in identifying functional areas and the modulation of C-type lectin receptor (CLR) activity, thereby contributing to the development of its associated functions [19]. CL-K1 is capable of binding to the carbohydrate moieties present on pathogenic microorganisms, thereby triggering the activation of MASPs. This activation subsequently initiates the lectin pathway of complement activation, leading to the stimulation of immune responses and inflammatory processes [17,20,22]. Additionally, CL-K1 takes part in the innate immunity as a mediator, exhibiting antibacterial and modulatory roles in uropathogenic *Escherichia coli* (UPEC) infection [23]. It acts as an initiator of the complement cascade and a regulator of T-cell reactions, providing insights into the underlying mechanisms that regulate the immunogenic characteristics of retinal pigment epithelium cells [24]. Mutations in the CL-K1 or MASP-1/3 genes may affect normal body development, resulting in a 3MC syndrome which encompasses the overlapping Carnevale, Mingarelli, Malpuech, and Michels syndromes [21,25]. Furthermore, recent studies have reported that CL-K1 plays a significant role in cancer cell proliferation and tumor growth. It is considered as a crucial promoter of tumor growth and a potential target for cancer treatment [26].

Nile tilapia (*Oreochromis niloticus*) is a prominent fish species in global aquaculture, with China being the leading producer [27]. Tilapia farming plays a crucial role in agricultural and rural economic development, contributing to increased income for farmers and a reliable source of high-quality protein. However, the industry has recently faced significant challenges due to the outbreak of diseases, particularly caused by the pathogen *S. agalactiae*. These outbreaks have resulted in substantial economic losses for the aquaculture sector [28,29]. Therefore, it is imperative to comprehend the defense mechanisms against infectious bacterial diseases in Nile tilapia to prevent future outbreaks. In aquatic animals, various lectins have been studied across different species, and they are known to play an important role in immune defense. Lectins such as MBL [30,31], L-rhamnose-binding lectin (RBL-L), and CL-K1 have been reported to have significant immune defense functions [30,31,32,33]. However, information regarding CL-K1 in teleost fish is limited. Previous studies have only identified CL-K1 in black rockfish, where it functions as a recognition molecule in pathogen resistance [34]. Additionally, the expression profiles of CL-K1 have been examined, and it has been indicated that the prokaryotic recombinant CL-K1 protein from Nile tilapia presents potent antibacterial activity in vitro [33]. Nevertheless, whether CL-K1 becomes involved in the host defense against pathogenic bacteria infection remains unclear in teleost fish.

In this study, we investigated the functional characteristics of *On*CL-K1 in the host defense against *S. agalactiae*. The impact of *On*CL-K1 on the bacterial load in various organs of infected tilapia was assessed using plate counting. Furthermore, the study examined the levels of *IL-6*, *IL-10*, *IL-8*, and *MIF* gene expression in different tissues and organs following artificial infection of tilapia, as well as the extent of tissue damage. The immune protective effect of *On*CL-K1 in tilapia was evaluated by calculating the statistical survival rate. The findings indicate that *On*CL-K1 plays a vital role in enhancing tilapia resistance to pathogen infection and significantly inhibits pathogen proliferation within the body.

## 2. Results

### 2.1. OnCL-K1 Promotes Bacterial Clearance

**Figure 1:**
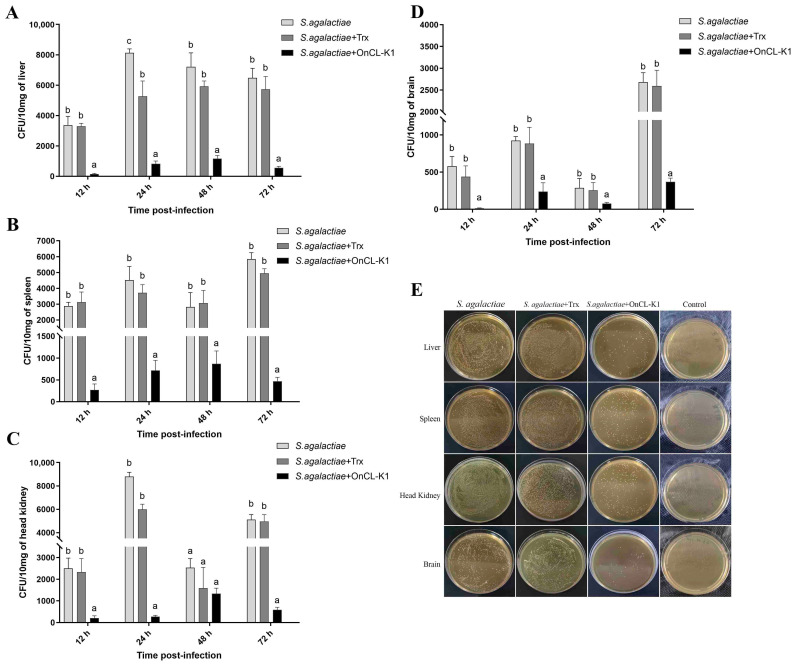
*On*CL-K1 suppresses bacterial propagation in vivo. Nile tilapia were infected with *S. agalactiae* (3 × 10^6^ CFU/fish) in the presence or absence of *On*CL-K1 and Trx. The control group received the same volume of PBS. Bacterial counts were determined in the liver (**A**), spleen (**B**), head kidney (**C**), and brain (**D**) at different time points post-infection. (**E**) Part of the coating plate picture display. The error bars represent standard deviation (*n* = 3) and significant difference is indicated by different letters (a, b, c) (*p* < 0.05).

To investigate the effect of *On*CL-K1 on the clearance of bacteria, tilapia were subjected to injections of *S. agalactiae* and *S. agalactiae*, along with *On*CL-K1 or Trx, and PBS. Bacterial counts were assessed in the liver, spleen, anterior kidney, and brain at 12, 24, 48, and 72 h post-infection. The findings revealed that the number of bacterial colonies in the liver, spleen, anterior kidney, and brain tissues of tilapia in the *On*CL-K1 group was significantly lower compared to the *S. agalactiae* group and the Trx group (Figure 1E). No *S. agalactiae* were detected in the blank control group. In the liver tissues, the bacterial load of the *On*CL-K1 group decreased significantly within 72 h after infection. At the 12 h time point post-challenge, the bacterial load of the *On*CL-K1 group was 24.1-fold lower when compared to the *S. agalactiae* infection group, and 23.6 times lower than that in the control protein Trx group (Figure 1A). In the spleen, the bacterial load in the *On*CL-K1 supplemented group decreased significantly within 72 h after infection, with no significant difference observed between the *S. agalactiae*-infected group and the Trx-supplemented group. At 12 h post-infection, the bacterial load in the *On*CL-K1 group was 10.7 times lower than that in the *S. agalactiae* group and 11.6 times lower than that in the control protein Trx group (Figure 1B). In the head kidney tissues, the bacterial load of the *On*CL-K1 group decreased significantly within 24 h after infection. At 24 h post-infection, the bacterial load in the *On*CL-K1 group was 32.5 times lower than that of the *S. agalactiae* group and 22.1 times lower than that of the control protein Trx group (Figure 1C). However, there was no significant difference among control groups and the *On*CL-K1 group (Figure 1C). In the brain, the bacterial load in the *On*CL-K1-supplemented group decreased significantly by more than five times compared to both the control protein Trx group and the *S. agalactiae* group within 72 h after infection (Figure 1D). At 24 h post-infection, the bacterial load of the *On*CL-K1 group was 3.9 times lower than that of the *S. agalactiae* group and 3.7 times lower than that of the control protein Trx group (Figure 1D).

### 2.2. Different Tissue Expression of OnCL-K1 after Infection In Vivo

**Figure 2:**
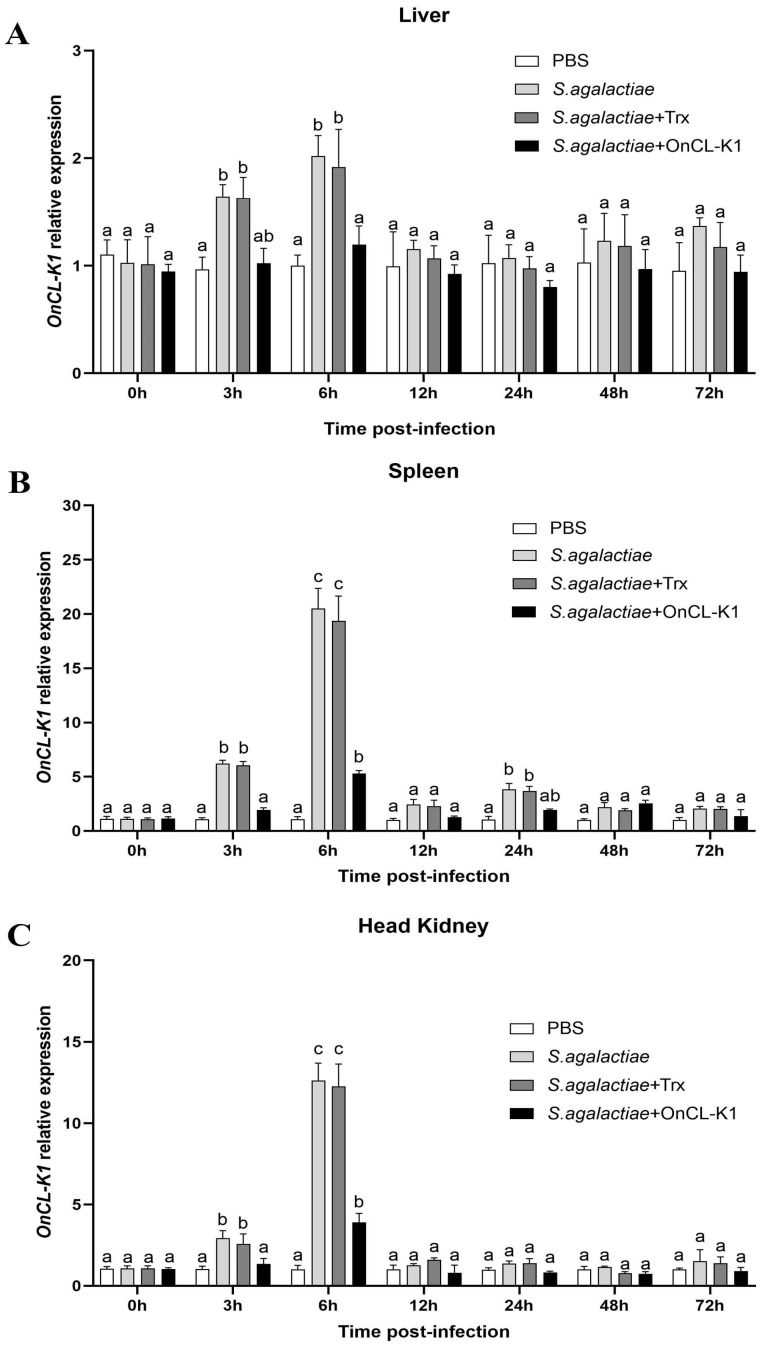
*OnCL-K1* expression at the mRNA level in different tissues and organs after *S. agalactiae* infection. The expression levels of *OnCL-K1* in liver (**A**), spleen (**B**), and head kidney (**C**) were determined using qRT-PCR as relative to *β*-actin at different time points post-infection with *S. agalactiae* in the presence or absence of *On*CL-K1 (*n* = 3). Values are shown as means ± SD and significant difference is marked by different letters (a, b, c) (*p* < 0.05).

To examine the effect of co-stimulation with *S. agalactiae* and CL-K1 on the level of *On*CL-K1 in various organs, qRT-PCR was employed to assess the variations following challenges with *S. agalactiae*, *S. agalactiae* plus *On*CL-K1, or the carrier protein Trx. In the liver tissue, the mRNA level of *On*CL-K1 reached the maximum at 6 h after infection. However, the group supplemented with *On*CL-K1 exhibited significantly lower expression of *On*CL-K1 compared to the group supplemented with *S. agalactiae* or Trx (Figure 2A). Similarly, both in the anterior kidneys or spleen, CL-K1 displayed a notable increase in expression at 6 h post-infection after challenges with *S. agalactiae*, *S. agalactiae* plus *On*CL-K1, or Trx. Nevertheless, the group supplemented with *On*CL-K1 demonstrated a notable decrease in CL-K1 expression compared to the groups of subjects challenged with *S. agalactiae* or Trx (Figure 2B,C).

### 2.3. Impacts of OnCL-K1 on Inflammatory and Migration Reactions In Vivo

**Figure 3:**
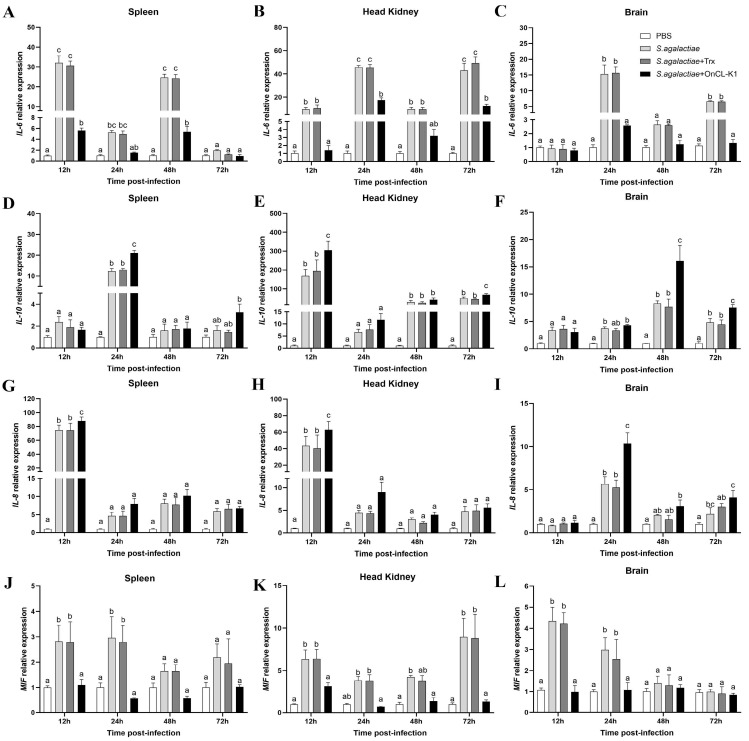
*On*CL-K1 reduces inflammation in vivo. The levels of *IL-6* (**A**–**C**), *IL-10* (**D**–**F**), *IL-8* (**G**–**I**), and *MIF* (**J**–**L**) in spleen, head kidney, and brain, respectively, were assessed using qRT-PCR as relative to *β*-actin at different time points post-infection with *S. agalactiae* in the presence or absence of *On*CL-K1 (*n* = 3). Values are shown as means ± SD and significant difference is marked by different letters (a, b, c) (*p* < 0.05).

To examine the impacts of *IL-6*, *IL-10*, *IL-8*, and *MIF* expression in the spleen, anterior kidney, and brain tissue of tilapia following infection with *S. agalactiae*, tilapia were challenged with *S. agalactiae*, *S. agalactiae* plus *On*CL-K1, or Trx, and PBS, respectively. In the spleen, the levels of *IL-6* (at 12 h, 24 h, and 48 h) and *IL-10* (at 24 h) expression were significantly up-regulated following *S. agalactiae* infection. However, the level of *IL-6* expression in the *On*CL-K1 group exhibited a substantial reduction when compared to both the *S. agalactiae* and Trx groups, while the level of *IL-10* expression was significantly higher compared to that in the *S. agalactiae* and Trx groups (Figure 3A,D). Additionally, the levels of *IL-8* (at 12 h) and *MIF* (at 12 h and 24 h) were also significantly increased. Furthermore, the level of *IL-8* expression in the *On*CL-K1 group exhibited a statistically significant increase compared to both *S. agalactiae* and Trx groups, while the expression of MIF of *On*CL-K1 group was significantly lower than that in the *S. agalactiae* and Trx groups (Figure 3G,J). Similarly, in the head kidney, the levels of *IL-6* and *IL-10* were significantly up-regulated. However, the level of *IL-6* expression in the *On*CL-K1 group was statistically significantly lower compared to both the *S. agalactiae* and Trx groups, while the level of *IL-10* was significantly higher than that in the *S. agalactiae* group and Trx group (Figure 3B,E). The expression levels of *IL-8* (at 12 h) and *MIF* (at 12 h and 72 h) were also significantly increased, with the level of *IL-8* expression of the *On*CL-K1 group being a notable increase compared to both the *S. agalactiae* and Trx groups, while the level of *MIF* was significantly lower compared to the *S. agalactiae* and Trx groups (Figure 3H,K). In the brain tissue, the levels of *IL-6* (at 24 and 72 h) and *IL-10* (at 48 h) were significantly up-regulated. However, the level of *IL-6* expression in the *On*CL-K1 group was significantly lower than that in both the *S. agalactiae* and Trx groups, while the level of *IL-10* expression was significantly higher compared to the *S. agalactiae* and Trx groups (Figure 3C,F). The levels of *IL-8* (at 24 h) and *MIF* (at 12 h and 24 h) were also significantly increased. In the *On*CL-K1 group, the level of *IL-8* expression was significantly higher compared to the *S. agalactiae* and Trx groups, while the expression level of *MIF* was significantly lower than that in the *S. agalactiae* and Trx groups (Figure 3I,L). In conclusion, *On*CL-K1 can reduce the level of the pro-inflammatory factor *IL-6* and migration inhibitor *MIF* and promote the level of anti-inflammatory factor *IL-10* and pro-inflammatory factor *IL-8* in the spleen, anterior kidney, and brain tissues.

### 2.4. Histopathological Examination

**Figure 4:**
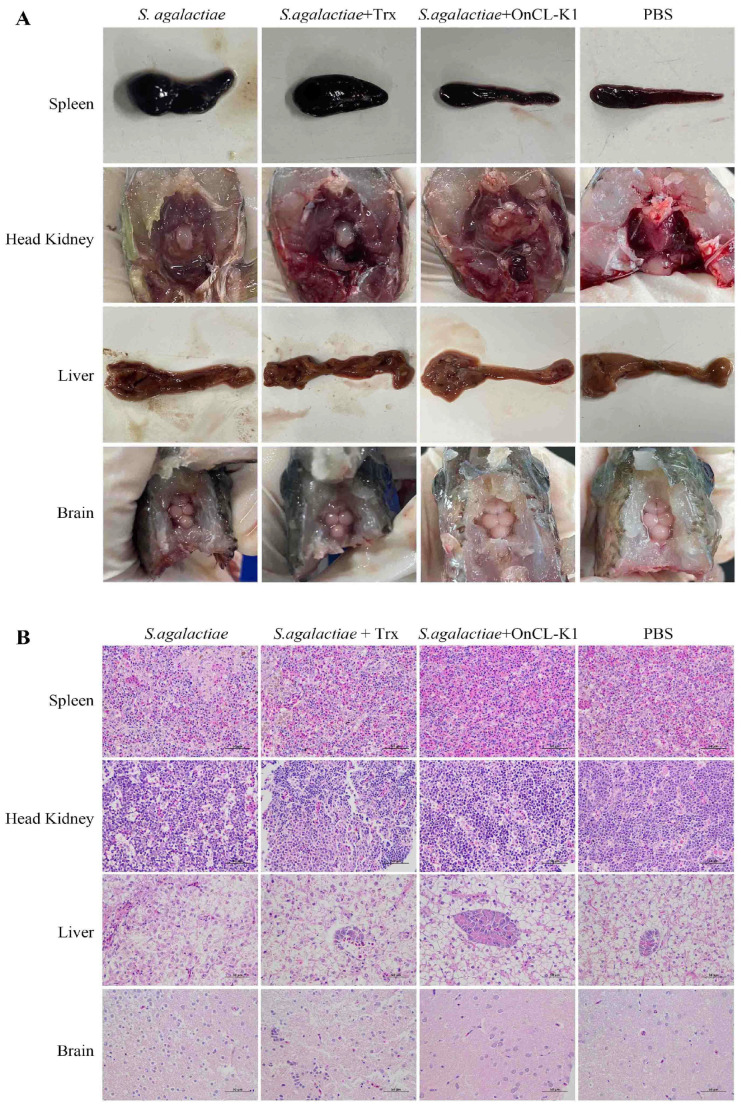
*On*CL-K1 reduces tissue lesions in Nile tilapia after *S. agalactiae* infection. Nile tilapia were infected with *S. agalactiae* (3 × 10^6^ CFU/fish) in the presence or absence of *On*CL-K1 and Trx. (**A**) Histopathological observation of spleen, head kidney, liver, and brain at 24 h post-infection. (**B**) H&E staining of spleen, head kidney, liver, and brain sections at 24 h post-infection. Scale bar, 50 μm.

The tissue or organ undergoes damage following infection with *S. agalactiae*. Upon gross observation at 24 h post-infection, Nile tilapia in the *S. agalactiae* and Trx groups exhibited significant swelling in the spleen and anterior kidney. Concurrently, they also showed significant hepatic congestion and meningeal hemorrhage. However, the spleen, anterior kidney, liver, and brain tissue of Nile tilapia in the *On*CL-K1 group did not show significant enlargement and hemorrhage, and their corresponding tissue morphology resembled that of the control group (Figure 4A).

The findings of H&E staining of tissue sections showed the following characteristics in the liver tissues at 24 h post-infection, compared to the control group: disordered liver cord structure, loose and unclear outline, edema of liver cells, vacuole degeneration, and the presence of vacuoles of varying sizes in the cytoplasm. Some hepatocytes exhibited lightly stained cytoplasm and nucleus, dissolution, and disappearance, leaving only the cell outline. Dissolved and lost cells fused together to form a fuzzy, unstructured necrotic lesion (Figure 4B). In the spleen, there were scattered deposits of hemosiderin, reduction in white pulp volume, lymphocytopenia, edema of splenic parenchymal cells, nucleus contraction, transparent cytoplasm vacuoles, and amyloid degeneration of plasma protein exudation. The epithelial tissue of renal tubules in the head renal tissue showed swelling and disarray, with some epithelial cells undergoing necrosis and infiltration, and the interstitium being infiltrated by inflammatory cells. Edematous dilatation of renal sacs and severe dilatation and congestion of glomerular capillaries were observed (Figure 4B). The brain stroma exhibited severe edema with numerous spaces, displaying a spongy appearance. Nerve-cell degeneration and necrosis were evident, and Purkinje’s cells were structureless, with decreased and dissolved nuclear chromatin (Figure 4B). The liver, spleen, anterior kidney, and brain tissue of fish supplemented with the control protein Trx exhibited similar characteristics. Apart from the *On*CL-K1 group, the liver cord structure of Nile tilapia was clear, hepatocytes did not show obvious edema, and the cytoplasm and nucleus structures of hepatocytes were distinct (Figure 4B). There was no significant deposit of hypoxanthine in the spleen, and the distribution of white and red pulp was reasonable, with no apparent edema in spleen parenchymal cells. The tissue structure of the head kidney was orderly, and no significant swelling was observed. The brain structure resembled that of the control Trx group (Figure 4B).

### 2.5. OnCL-K1 Improved the Survival Rate of Nile Tilapia Infected with S. agalactiae

**Figure 5:**
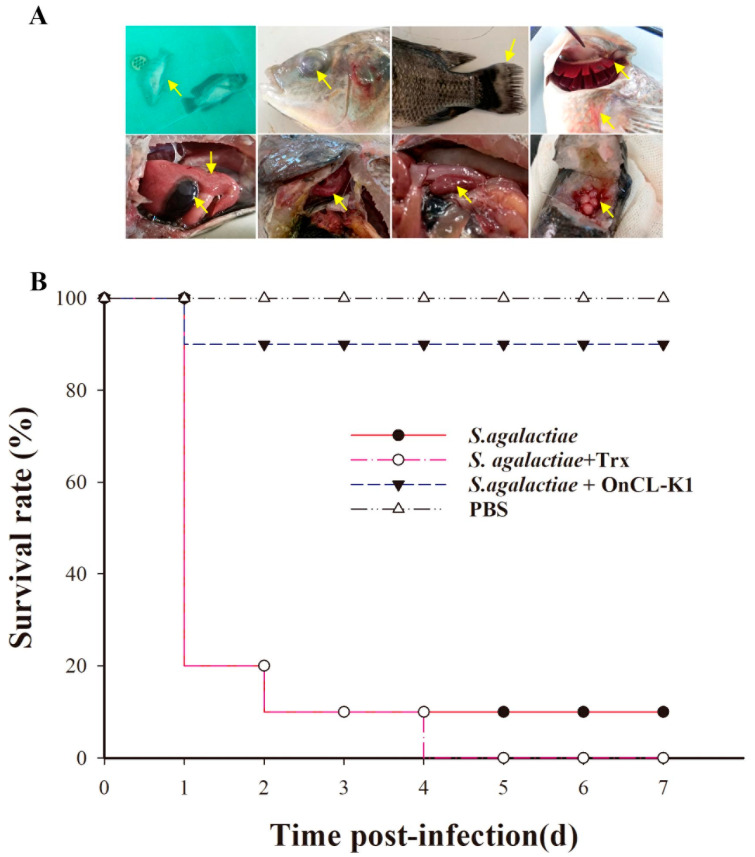
Effects of *On*CL-K1 on the survival rate of *S. agalactiae*-infected Nile tilapia. Fish were infected with *S. agalactiae* (3 × 10^7^ CFU/fish) in the presence or absence of *On*CL-K1 and Trx. The control group fish received the same volume of PBS (20 fish for each group). (**A**) Morphological observation of Nile tilapia after *S. agalactiae* infection. The yellow arrows indicate the characteristic symptoms of Nile tilapia streptococcus disease, including swimming imbalance, bulging eyes, fin rot, congestion of gill cap (Figure 5A top line from left to right, respectively), swelling of liver and gallbladder, anterior kidney, and spleen, and congestion in the brain cavity (Figure 5A below line from left to right, respectively). (**B**) The survival rate of different groups by *S. agalactiae* infected within the presence or absence of *On*CL-K1 and Trx.

To investigate the impact of *On*CL-K1 on the survival rate of fish infected with *S. agalactiae*, healthy Nile tilapia were artificially infected with *S. agalactiae* through intraperitoneal injection. Three additional groups were included: one group received *On*CL-K1, another group received Trx protein, and the third group received PBS (the control group). The results demonstrated that tilapia exhibited characteristic symptoms of Nile tilapia streptococcus disease, including swimming imbalance, blackening of body color, fin rot, bulging eyes, congestion of gill cap, swelling of liver, gallbladder, anterior kidney, and spleen, and congestion in the brain cavity. Further, *S. agalactiae* was isolated from the liver, spleen, head kidney, and brain tissues of infected fish, but not from the control group (Figure 5A). This indicates that *S. agalactiae* infection was the primary cause of Nile tilapia mortality. Moreover, survival statistics revealed that *On*CL-K1 significantly enhanced the survival rate of Nile tilapia following *S. agalactiae* infection (90%), whereas Trx protein had no significant impact on the survival rate of Nile tilapia after infection (Figure 5B).

## 3. Discussion

Anti-infective immunity refers to the collective physiological and pathological immune responses mounted by the immune system to identify and eliminate pathogens [34]. When hosts are invaded by pathogens, infection occurs, and the host’s immune system recognizes the pathogens, subsequently triggering innate and adaptive immune responses [35]. Lectins, such as CL-K1, are crucial in the anti-infection process as they participate in pathogen recognition, activation of the complement pathway, agglutination, opsonophagocytosis, and other functions. CL-K1, a multifunctional pattern recognition lectin, has been isolated from Nile tilapia and has demonstrated significant pathogen agglutination capabilities [33]. In this research, we examined the role of CL-K1 in eliminating bacterial pathogen in Nile tilapia. Our results demonstrate that CL-K1 effectively suppresses the growth of *S. agalactiae* in Nile tilapia and improves the survival rate of tilapia during bacterial infection.

*Streptococcus agalactiae* has the ability to colonize the gastrointestinal lumen of Nile tilapia. Some bacteria can survive and multiply within macrophages, evading or infecting the host’s immune defense system. This leads to a rapid proliferation of *S. agalactiae* in the host, causing acute systemic infection through the production of toxins [36]. Lectins play an important role in binding to pathogens and eliminating them by activating complement, opsonophagocytosis, and agglutination [32,37,38]. CL-K1, a multifunctional C-type lectin, has been identified in Nile tilapia (*Oreochromis niloticus*), half-smooth tongue sole (*Cynoglossus semilaevis*), and other fish. It has been found to have an agglutination effect on pathogens [33,39]. The study [34] demonstrated that *On*CL-K1 from Nile tilapia strongly agglutinate *S. agalactiae* and *Aeromonas hydrophila*, effectively inhibiting their growth in vitro. Furthermore, the study discovered that *On*CL-K1 significantly inhibits the growth of pathogens in the liver, spleen, anterior kidney, and brain tissue of fish infected with *S. agalactiae*. The liver, which serves as both a digestive and immune organ, plays a vital role in resisting pathogen infection and is crucial for the overall health and growth of the fish. Previous reports have showed that the natural infection of Nile tilapia with *S. agalactiae* leads to degeneration and swelling of vascular endothelial cells in the liver tissue, resulting in thrombosis and local hepatic infarction [40]. The spleen, the primary immune and hematopoietic organ in fish, forms multiple melanin-macrophage centers around blood vessels and in the parenchyma as a defense mechanism against invading *S. agalactiae*. However, *S. agalactiae* can invade the spleen and rapidly multiply, leading to degenerative pathological changes such as inflammatory bleeding and lymphocyte necrosis, ultimately causing splenic septicemia [40]. The head kidney, similar to mammalian bone marrow, is a crucial hematopoietic organ in fish [41,42]. At the same time, the head kidney functions as a secondary lymphoid organ in teleost fish, capable of phagocytosing and eliminating pathogens, as well as releasing pro-inflammatory factors to activate the body’s immune response [43,44]. The head kidney of Nile tilapia primarily consists of hematopoietic and lymphoid tissue, containing a substantial number of mature lymphocytes and melanin macrophage centers. These components are crucial immune organs in Nile tilapia [45]. The fish brain acts as the central nervous system’s high-level center, and it is protected by the blood–brain barrier (BBB) [40]. *S. agalactiae* can breach the blood–brain barrier of Nile tilapia, entering the central nervous system and causing abnormal clinical symptoms such as disorientation and swimming imbalance [40]. In experiments involving intraperitoneal injection, the infection rate of *S. agalactiae* was rapid, with a significant presence detected in the liver and anterior kidney within 3 h of infection. Within 6~8 h, the amount of *S. agalactiae* in the brain was significantly increased [46]. This study also observed a substantial presence of *S. agalactiae* in the liver, spleen, anterior kidney, and brain tissues of fish within 12 to 72 h post-infection. The addition of *On*CL-K1 significantly decreased the bacterial load of *S. agalactiae* in various organs, while significantly increasing the survival rate of Nile tilapia (90%). In our previous study [33], we demonstrated that the recombinant *On*CL-K1 protein has the ability to bind to and agglutinate *S. agalactiae*, similar to other lectins found in tilapia such as MBL and L-rhamnose-binding lectin [31,32]. Furthermore, the (r)*On*CL-K1, acting as an opsonin, enhances the phagocytosis of *S. agalactiae* by tilapia macrophages [33], indicating its role in the clearance of the pathogen *S. agalactiae*. These findings suggest that *On*C-K1 may play a crucial role in influencing pathogen colonization, thereby facilitating pathogen clearance in tilapia and reducing the risk of disease.

*S. agalactiae* infection in Nile tilapia can elicit a robust inflammatory response [47]. Upon infection, *S. agalactiae* binds to pattern recognition receptors on host cells, initiating an innate immune response [48]. These receptors include phagocytic receptors (e.g., mannose receptors, scavenger receptors, C-type lectin receptors) and membrane/cytoplasmic signal receptors (e.g., TLR, NLR, RLR, CDS), which mediate pathogen phagocytosis, activate inflammatory genes via signal transduction, and secrete pro-inflammatory factors and anti-inflammatory factors. IL-6 is a pro-inflammatory factor produced during tissue injury and infection, playing a vital role in immune and inflammatory processes, as well as cellular proliferation and differentiation [49]. IL-10, a recognized immunosuppressive cytokine, acts as a homologous dimer to inhibit antigen presentation and cytokine synthesis in macrophages [50]. IL-8, a constituent of the chemokine family, exhibits potent chemotactic properties, promoting cell activation, adhesion, and inflammation regulation [51]. MIF exerts inhibitory effects on the migration of monocytes and macrophages. It achieves this by engaging in interaction with different cytokines, inflammatory factors, and substances, thereby modulating the process of inflammation [52,53]. In this study, the levels of *IL-6*, *IL-10*, *IL-8*, and *MIF* expression in the spleen, anterior kidney, and brain tissues of Nile tilapia significantly increased upon *S. agalactiae* infection, while the expression levels of *IL-6* and *MIF* in corresponding tissues of Nile tilapia co-stimulated with *On*CL-K1 were highly reduced. The expression of *IL-10* and *IL-8* in spleen, anterior kidney, and brain tissue were up-regulated at different time points after infection. However, the peak time for upregulation or downregulation of *IL-6* and *IL-10*, or *IL-8* and *MIF* cytokines, was not consistent. Since IL-6, IL-8, and MIF are cytokines that are crucial in the inflammation and leukocyte migration process for host defense, typically the expression patterns of these genes are similar. However, in the group infected with *S. agalactiae* and co-stimulated with *On*CL-K1 protein, the expression pattern of *IL-8* differed from that of *IL-6* and *MIF*. Further investigation is needed to understand the regulatory mechanism behind this difference in cytokine expression patterns. Additionally, histopathological analysis revealed varying degrees of damage and inflammatory cell infiltration in the liver, spleen, anterior kidney, brain, and other tissues of fish following *S. agalactiae* infection, while *On*CL-K1 significantly alleviated the damage and inflammation symptoms. The findings indicate that *On*CL-K1 may become involved in the regulation of inflammation and the migration of phagocytes during the anti-infection process. However, more research is needed to understand its specific regulatory mechanism.

Collectively, our findings demonstrate that *On*CL-K1 has a significant bactericidal effect on pathogens in various organs of Nile tilapia, including the liver, spleen, anterior kidney, and brain tissue, following *S. agalactiae* infection. Moreover, *On*CL-K1 effectively inhibits pathogen replication in Nile tilapia, leading to a notable improvement in the survival rate of infected individuals. Additionally, *On*CL-K1 exhibits the ability to modulate the levels of inflammatory factors, such as *IL-6* and *IL-10*, as well as chemotactic migration factors, including *IL-8* and *MIF*, in Nile tilapia post-infection with *S. agalactiae*. This regulatory function contributes to the reduction of tissue damage and inflammatory response in fish infected with *S. agalactiae*. Our study highlights the potential of *On*CL-K1 as a crucial collagen lectin involved in host innate immune defense, exerting antibacterial effects during the anti-infection process.

## 4. Materials and Methods

### 4.1. Fish Rearing

Nile tilapia (*Oreochromis niloticus*) specimens were raised at the Guangdong Tilapia Breeding Farm (Guangzhou, China), with an average weight of 50 ± 5 g. Before conducting the experiments, the fish were acclimated in a recirculating water system at a temperature of 28 ± 2 °C for a duration of 2 weeks [32,33,34]. Additionally, the water used was city water that had been filtered biologically, dechlorinated, chemically balanced, and treated with UV light. The pH of the pool water was maintained between 7.0 and 8.0. Each day, 4% of the water was exchanged with fresh water, while 75% of the volume was recirculated through the biofilter every hour. The animal protocol underwent a thorough review and received approval form the University Animal Care and Use Committee at South China Normal University (approval number: SCNU-SLS-2022-007).

### 4.2. Preparation of OnCL-K1 Recombinant Protein

Tilapia recombinant CL-K1 protein was prepared and purified as previously described [33]. In detail, the sequence of *On*CL-K1 was cloned and linked with pET-32a expression plasmid. After being transferred into BL21 (DE3) *Escherichia coli*, the plasmid could increase with the proliferation of bacteria cultured in LB-ampicillin. Once the culture medium reached an O.D. 600 of 0.6–0.8, we added Isopropyl-β-D-thiogalactopyranpside (IPTG) to a final concentration of 1 mM and cultured the bacteria at 37 °C for 6 h. Subsequently, the culture medium was centrifuged at 3300× *g* for 30 min at 4 °C, and the cells were re-suspended in PBS. Lysozyme, dissolved in water to 10 mg/mL (Sigma-Aldrich, Shanghai, China), was added to the cells at a ratio of 1:100 and allowed to react for 3 h. Following the reaction, the cell mixture was disrupted using an Ultrasonic Processor (ShunmaTech, Guangzhou, China) to release the protein. The cell lysate was then centrifuged at 10,000 rpm for 30 min at 4 °C, and the resulting precipitate was re-suspended in Lysis Buffer (8 M Urea, 50 mM NaH_2_PO_4_, 300 mM NaCl, 10 mM imidazole, pH 8.0). Purification was carried out using Ni-NTA His Band Resin columns (Novagen, Darmstadt, Germany) according to the protocol [33]. The columns with nickel ions were balanced with 5 mL of Lysis Buffer, and the protein solution was added for full contact. After draining the liquid from the column, 15 mL of Wash Buffer (8 M Urea, 50 mM NaH_2_PO_4_, 300 mM NaCl, 20 mM imidazole, pH 8.0) and 5 mL of Elution Buffer (8 M Urea, 50 mM NaH_2_PO_4_, 300 mM NaCl, pH 8.0) with different concentrations of imidazole (20 mM, 40 mM, 60 mM, 200 mM, and 1 M) were sequentially added and collected to obtain the recombinant protein. The concentration of *On*CL-K1 protein was adjusted within an appropriate range by using PEG 20,000. Additionally, the Trx-Pet-32a (Trx) recombinant protein was also prepared as previously reported [33]. In brief, Trx-Pet-32a (Trx) recombinant protein is a kind of protein expressed from pET-32a vector, which could be also purified from Ni-Agarose His-Tagged Protein Purification column. The steps and conditions of its expression and purification followed the same method as (r)*On*CL-K1.

### 4.3. Immunization and Sample Collection

Nile tilapia specimens were intraperitoneally injected with 100 μL of *S. agalactiae* (3 × 10^6^ CFU/fish), *S. agalactiae* (3 × 10^6^ CFU/fish) plus *On*CL-K1 (30 μg/fish), or Trx (30 μg/fish) in PBS with a final concentration of 3 × 10^7^ CFU/mL. The blank control groups were injected with the same volume of PBS. At the time points of 12, 24, 48, and 72 h post-injection, liver, spleen, anterior kidney, and brain tissue samples were harvested and frozen using liquid nitrogen or fixed in tetramethylene for further analysis.

To assess the expression of *On*CL-K1, the tissue samples including liver, anterior kidney, and spleen were collected at 12, 24, 48, and 72 h post-injection from both the stimulation and blank control groups. Additionally, cytokine levels in the tissues of anterior kidney, spleen, and brain were measured at 12, 24, 48, and 72 h post-infection.

### 4.4. Bacterial Counts

The bacterial clearance assay was conducted following previously established protocols with some modifications. *S. agalactiae* was prepared in PBS at a concentration of 3 × 10^7^ CFU/mL. The cells were then treated with *On*CL-K1 and Trx, resulting in a final concentration of 300 μg/mL. The stimulation groups were injected with 100 μL *S. agalactiae*, *S. agalactiae* with *On*CL-K1, or *S. agalactiae* with Trx. The black control groups received an equivalent dose of PBS. At 12 h, 24 h, 48 h, and 72 h post-infection, liver, spleen, anterior kidney, and brain samples were collected. Each tissue sample was homogenized and mixed with PBS at the ratio of 1 mg/mL. The quantification of remaining bacteria was accomplished through inoculating the samples on brain heart infusion (BHI) agar plates. Each group consisted of five Nile tilapias, and the test was repeated in triplicate.

### 4.5. qRT-PCR Method for Detection of OnCL-K1 Gene Expression

The transcriptional profile of *OnCL-K1* in different organs from both the stimulation and blank control groups was assessed using qRT-PCR [33]. In this study, head kidney, spleen, brain, and liver were employed for further experiments. The polymerase chain reaction mixture consisted of a total volume of 20 μL, reagents and dosage followed the manual of the Hieff^®^ qPCR SYBR Mix (Yeasen Biotechnology). The PCR program included an initial denaturation step at 95 °C for 3 min, followed by denaturation at 95 °C for 15 s, annealing at 60 °C for 1 min, and then proceeding to step 2, which was repeated for a total of forty cycles. The quantification of *OnCL-K1* gene expression was conducted by normalizing the expression level to Nile tilapia *β*-actin [33,54,55], and further comparing the results to the respective control group to assess changes in gene expression. The level of *OnCL-K1* expression was determined using the 2^−ΔΔCt^ method in order to obtain relative expression values [56]. The quantitative expression data obtained in the study were then reported as the mean values ± standard deviation (SD).

### 4.6. Impacts of OnCL-K1 on Migration and Inflammation Response after Infection

Tissue samples were subjected to lysis using Trizol Reagent to extract RNA at specific time points of 12 h, 24 h, 48 h, and 72 h following bacterial infection. The mRNA levels of *IL-6*, *IL-10*, *IL-8*, and *MIF* were quantified using qRT-PCR. The primers in Table 1 utilized in this study were obtained from fellow researchers in our laboratory. The relative expressions of these four genes in Nile tilapia were normalized to that of Nile tilapia *β*-actin and compared to the respective control group to investigate changes in gene expression. The calculation means of relative expression levels and the presentation of quantitative expression data were the same as those in Section 2.4.

### 4.7. Histopathological Observation

For histopathological analysis, the liver, spleen, head kidney, and brain samples were harvested. These samples were injected with *S. agalactiae*, *S. agalactiae* plus *On*CL-K1, or Trx, and a control group received PBS injection. After 24 h, all tissues were fixed in a 4% paraformaldehyde solution. Paraffin sections were then prepared from the fixed tissues and subjected to H&E staining for histopathological observation and analysis. The paraffin sections and H&E staining were performed by Wuhan Seville Biotechnology Co., Ltd., (Wuhan, China).

### 4.8. Fish Survival Assay

For the survival assay, a cohort of 80 fishes were subjected to random allocation, resulting in the formation of four distinct groups, each consisting of 20 individuals. Nile tilapias were injected with 100 μL *S. agalactiae* (3 × 10^8^ CFU/mL) alone, or in combination with *On*CL-K1 (300 μg/mL) or Trx (300 μg /mL). The blank control group was administered an injection of an equal volume of PBS. Over a period of 7 days, the mortality rate of the fish was recorded at 24 h intervals. The survival rate was evaluated using the Kaplan–Meier method.

### 4.9. Statistical Analysis

The experiments were conducted in triplicate and statistical analyses were performed using GraphPad Prism 9. The data were subjected to one-way ANOVA analysis to determine statistical significance, which was defined as *p* < 0.05. The results were represented as mean ± standard error. Figures illustrating the survival rate were generated using Sigma Plot 14.0 software, and GraphPad Prism 9 software was employed for other data analyses in this study.

## Figures and Tables

**Table 1:** Primers used in this study.

Primers	Nucleotide Sequence (5′-3′)	Purpose
*β*-actin-F	CGAGAGGGAAATCGTGCGTGACA	Control
*β*-actin-R	AGGAAGGAAGGCTGGAAGAGGGC	Control
qCL-K1-F	AGGTTCTCGTGGTCCCAAAGG	qRT-PCR
qCL-K1-R	CCAAGTCGTCCCACATTACCAA	qRT-PCR
qIL-6-F	ACAGAGGAGGCGGAGATG	qRT-PCR
qIL-6-R	GCAGTGCTTCGGGATAGAG	qRT-PCR
qIL-8-F	GATAAGCAACAGAATCATTGTCAGC	qRT-PCR
qIL-8-R	CCTCGCAGTGGGAGTTGG	qRT-PCR
qIL-10-F	TGGAGGGCTTCCCCGTCAG	qRT-PCR
qIL-10-R	CTGTCGGCAGAACCGTGTCC	qRT-PCR
qMIF-F	CACATCAACCCTGACCAAAT	qRT-PCR
qMIF-R	GCCTGTTGGCAGCACC	qRT-PCR

## Data Availability

The data presented in this study are available from the corresponding author on reasonable request.

## References

[1] Diamond M.S., Kanneganti T.D. (2022). Innate immunity: The first line of defense against SARS-CoV-2. Nat. Immunol..

[2] Kumar H., Kawai T., Akira S. (2011). Pathogen recognition by the innate immune system. Int. Rev. Immunol..

[3] Akira S., Uematsu S., Takeuchi O. (2006). Pathogen recognition and innate immunity. Cell.

[4] Thaiss C.A., Zmora N., Levy M., Elinav E. (2016). The microbiome and innate immunity. Nature.

[5] Kaur B.P., Secord E. (2021). Innate Immunity. Immunol. Allergy Clin. N. Am..

[6] Vasta G.R., Nita-Lazar M., Giomarelli B., Ahmed H., Du S., Cammarata M., Parrinello N., Bianchet M.A., Amzel L.M. (2011). Structural and functional diversity of the lectin repertoire in teleost fish: Relevance to innate and adaptive immunity. Dev. Comp. Immunol..

[7] Doherty T.M., Arditi M. (2005). Innate immunity, Toll-like receptors and host response to infection. Pediatr. Infect. Dis. J..

[8] Xia X., You M., Rao X.J., Yu X.Q. (2018). Insect C-type lectins in innate immunity. Dev. Comp. Immunol..

[9] Robinson M.J., Sancho D., Slack E.C., LeibundGut-Landmann S., e Sousa C.R. (2006). Myeloid C-type lectins in innate immunity. Nat. Immunol..

[10] Viana J.T., Rocha R., Maggioni R. (2022). Structural and functional diversity of lectins associated with immunity in the marine shrimp *Litopenaeusvannamei*. Fish Shellfish Immunol..

[11] Vasta G.R., Ahmed H., Tasumi S., Odom E.W., Saito K. (2007). Biological roles of lectins in innate immunity: Molecular and structural basis for diversity in self/non-self recognition. Adv. Exp. Med. Biol..

[12] Vasta G.R., Quesenberry M.S., Ahmed H., O’Leary N. (2001). Lectins from tunicates: Structure-function relationships in innate immunity. Adv. Exp. Med. Biol..

[13] Ofek I., Crouch E., Keisari Y. (2000). The role of C-type lectins in the innate immunity against pulmonary pathogens. Adv. Exp. Med. Biol..

[14] Mayer S., Raulf M.K., Lepenies B. (2017). C-type lectins: Their network and roles in pathogen recognition and immunity. Histochem. Cell Biol..

[15] Raymond B., Neyrolles O., Rombouts Y. (2020). C-type lectins in immunity to lung pathogens. Curr. Top. Microbiol. Immunol..

[16] Liu Y., Liu J., Pang X., Liu T., Ning Z., Cheng G. (2015). The roles of direct recognition by animal lectins in antiviral immunity and viral pathogenesis. Molecules.

[17] Hansen S.W., Ohtani K., Roy N., Wakamiya N. (2016). The collectins CL-L1, CL-K1 and CL-P1, and their roles in complement and innate immunity. Immunobiology.

[18] Keshi H., Sakamoto T., Kawai T., Ohtani K., Katoh T., Jang S.J., Motomura W., Yoshizaki T., Fukuda M., Koyama S. (2006). Identification and characterization of a novel human collectin CL-K1. Microbiol. Immunol..

[19] Selman L., Hansen S. (2012). Structure and function of collectin liver 1 (CL-L1) and collectin 11 (CL-11, CL-K1). Microbiol. Immunol..

[20] Hansen S., Selman L., Palaniyar N., Ziegler K., Brandt J., Kliem A., Jonasson M., Skjoedt M.O., Nielsen O., Hartshorn K. (2010). Collectin 11 (CL-11, CL-K1) is a MASP-1/3-associated plasma collectin with microbial-binding activity. J. Immunol..

[21] Venkatraman G.U., Furze C.M., Gingras A.R., Yoshizaki T., Ohtani K., Marshall J.E., Wallis A.K., Schwaeble W.J., El-Mezgueldi M., Mitchell D.A. (2015). Molecular basis of sugar recognition by collectin-K1 and the effects of mutations associated with 3MC syndrome. BMC Biol..

[22] Mabrook M., Abd E.A., Ali Y.M., Hassan R. (2022). Inhibition of CL-11 reduces pulmonary inflammation in a mouse model of *Klebsiella pneumoniae* lung infection. Microb. Pathog..

[23] Yuan H., Gao Z., Lu X., Hu F. (2021). Role of collectin-11 in innate defence against uropathogenic *Escherichia coli* infection. Innate Immun..

[24] Fanelli G., Romano M., Lombardi G., Sacks S.H. (2023). Soluble collectin 11 (CL-11) acts as a immunosuppressive molecule potentially used by stem cell-derived retinal epithelial cells to modulate T cell response. Cells.

[25] Rooryck C., Diaz-Font A., Osborn D.P., Chabchoub E., Hernandez-Hernandez V., Shamseldin H., Kenny J., Waters A., Jenkins D., Al Kaissi A. (2011). Mutations in lectin complement pathway genes COLEC11 and MASP1 cause 3MC syndrome. Nat. Genet..

[26] Wang J.X., Cao B., Ma N., Wu K.Y., Chen W.B., Wu W., Dong X., Liu C.-F., Gao Y.-F., Diao T.-Y. (2023). Collectin-11 promotes cancer cell proliferation and tumor growth. JCI Insight..

[27] Yuan Y., Yongming Y., Yunyun D., Zongli Z., Yunchong G., Yiqun Y. (2020). Technical efficiency of different farm sizes for tilapia farming in China. Aquac. Res..

[28] Liu L., Lu D., Xu J., Luo H., Li A. (2019). Development of attenuated erythromycin-resistant *Streptococcus agalactiae* vaccine for tilapia (*Oreochromis niloticus*) culture. J. Fish Dis..

[29] Phuoc N.N., Linh N.T.H., Crestani C., Zadoks R.N. (2021). Effect of strain and environmental conditions on the virulence of *Streptococcus agalactiae* (Group B *Streptococcus*; GBS) in red tilapia (*Oreochromis* sp.). Aquaculture.

[30] Lei L., Yunfei D. (2020). Antimicrobial activity of mannose binding lectin in grass carp (*Ctenopharyngodon idella*) in vivo and in vitro. Fish Shellfish Immunol..

[31] Mu L., Yin X., Wu H., Lei Y., Han K., Mo J., Guo Z., Li J., Ye J. (2020). Mannose-binding lectin possesses agglutination activity and promotes opsonophagocytosis of macrophages with calreticulin interaction in an early vertebrate. J. Immunol..

[32] Mu L., Yin X., Bai H., Li J., Wu H., Qi W., Wang B., Ye J. (2021). Expression and functional characterization of an L-rhamnose-binding lectin from Nile tilapia (*Oreochromis niloticus*) in host defense against bacterial infection. Aquaculture..

[33] Mu L., Yin X., Bian X., Wu L., Yang Y., Wei X., Guo Z., Ye J. (2018). Expression and functional characterization of collection-K1 from Nile tilapia (*Oreochromis niloticus*) in host innate immune defense. Mol. Immunol..

[34] Yang T.Z., Zhu Q., Xue T., Cao M., Fu Q., Yang N., Li C., Huo H.J. (2022). Identification and functional characterization of CL-11 in black rockfish (*Sebastes schlegelii*). Fish Shellfish Immunol..

[35] Garraud O., Cognasse F., Hamzeh-Cognasse H., Pozzetto B. (2016). Platelets and their immune role in anti-infective immunity. Future Microbiol..

[36] Su Y., Feng J., Liu C., Li W., Xie Y., Li A. (2017). Dynamic bacterial colonization and microscopic lesions in multiple organs of tilapia infected with low and high pathogenic *Streptococcus agalactiae* strains. Aquaculture.

[37] Mu L., Yin X., Xiao Y., Bian X., Yang Y., Wu L., Ye J. (2018). A C-type lectin (CL11X1-like) from Nile tilapia (*Oreochromis niloticus*) is involved in host defense against bacterial infection. Dev. Comp. Immunol..

[38] Huang L., Bai L., Chen Y., Wang Q., Sha Z. (2019). Identification, expression profile and analysis of the antimicrobial activity of collectin 11 (CL-11, CL-K1), a novel complement-associated pattern recognition molecule, in half-smooth tongue sole (*Cynoglossus semilaevis*). Fish Shellfish Immunol..

[39] Zamri-Saad M., Amal M.N.A., Siti-Zahrah A. (2010). Pathological changes in red tilapias (*Oreochromis* spp.) naturally infected by *Streptococcus agalactiae*. J. Comp. Pathol..

[40] Huang J. (2012). Study on Etiology, Pathology of Tilapias *Streptococcus agalactiae* Disease and on the Function of *cpsE* Gene. Ph.D. Thesis.

[41] Rombout J.H.W.M., Huttenhuis H.B.T., Picchietti S., Scapigliati G. (2005). Phylogeny and ontogeny of fish leucocytes. Fish Shellfish Immunol..

[42] Rasighaemi P., Basheer F., Liongue C., Ward A.C. (2015). Zebrafish as a model for leukemia and other hematopoietic disorders. J. Hematol. Oncol..

[43] Franz V., Alin C., Rodolfo A., Margarita I.C. (2013). High density lipoproteins down-regulate transcriptional expression of pro-inflammatory factors and oxidative burst in head kidney leukocytes from rainbow trout, *Oncorhynchus mykiss*. Fish Shellfish Immunol..

[44] Miriam F., Elisabeth H., Pedro A., Kai K.L., Mari M. (2013). Cytokine gene expression and prostaglandin production in head kidney leukocytes isolated from Atlantic cod (*Gadus morhua*) added different levels of arachidonic acid and eicosapentaenoic acid. Fish Shellfish Immunol..

[45] He J. (2018). Histopathology of the Head Kidney in Tilapia Infected by *Streptococcus agalactiae*. Ph.D. Thesis.

[46] Guo F. (2018). The Mechanism of Influence of Different Water Temperature on *Streptococcus agalactiae* Infection in Tilapia. Ph.D. Thesis.

[47] Pattanapon K., Nopadon P., Ikuo H., Channarong R. (2014). Increasing of temperature induces pathogenicity of *Streptococcus agalactiae* and the up-regulation of inflammatory related genes in infected Nile tilapia (*Oreochromis niloticus*). Vet. Microbiol..

[48] Zhang X., Huang Y., Cai J., Jian J., Wang B. (2023). *Effect of miRNA*-155-targeted *SOCS5* gene on inflammatory response of brain astrocytes of *Oreochromis niloticus* induced by *Streptococcus agalactiae*. J. Guangdong Ocean Univ..

[49] Tanaka T., Narazaki M., Kishimoto T. (2014). IL-6 in inflammation, immunity, and disease. Cold Spring Harb. Perspect. Biol..

[50] Saraiva M., O’Garra A. (2010). The regulation of IL-10 production by immune cells. Nat. Rev. Immunol..

[51] Li A., Dubey S., Varney M.L., Dave B.J., Singh R.K. (2003). IL-8 directly enhanced endothelial cell survival, proliferation, and matrix metalloproteinases production and regulated angiogenesis. J. Immunol..

[52] Sumaiya K., Langford D., Natarajaseenivasan K., Shanmughapriya S. (2022). Macrophage migration inhibitory factor (MIF): A multifaceted cytokine regulated by genetic and physiological strategies. Pharmacol. Ther..

[53] Kang I., Bucala R. (2019). The immunobiology of MIF: Function, genetics and prospects for precision medicine. Nat. Rev. Rheumatol..

[54] Mu L., Yin X., Qi W., Li J., Bai H., Chen N., Yang Y., Wang B., Ye J. (2022). An l-rhamnose-binding lectin from Nile tilapia (*Oreochromis niloticus*) possesses agglutination activity and regulates inflammation, phagocytosis and respiratory burst of monocytes/macrophages. Dev. Comp. Immunol..

[55] Yang L., Bu L., Sun W., Hu L., Zhang S. (2014). Functional characterization of mannose-binding lectin in zebrafish: Implication for a lectin-dependent complement system in early embryos. Dev. Comp. Immunol..

[56] Livak K.J., Schmittgen T.D. (2001). Analysis of relative gene expression data using real-time quantitative PCR and the 2^−ΔΔCT^ Method. Methods.

